# Informing Hospital Physician Well-Being Interventions in Europe and the US

**DOI:** 10.1001/jamanetworkopen.2025.44067

**Published:** 2025-11-17

**Authors:** Linda H. Aiken, Walter Sermeus, Martin McKee, Reinhard Busse, Herbert Smith, Claudia B. Maier, Jonathan Drennan, Simon Dello, Dorothea Kohnen, Matthew D. McHugh, Karen B. Lasater

**Affiliations:** 1 Center for Health Outcomes and Policy Research, University of Pennsylvania, Philadelphia; 2 Leonard Davis Institute of Health Economics, University of Pennsylvania, Philadelphia; 3 Leuven Institute for Healthcare Policy, KU Leuven, Leuven, Belgium; 4 London School of Hygiene and Tropical Medicine, London, United Kingdom; 5 Department of Health Care Management, Berlin University of Technology, Berlin, Germany; 6 Population Studies Center, University of Pennsylvania, Philadelphia; 7 Department of Sociology, University of Pennsylvania, Philadelphia; 8 Bielefeld University, Bielefeld, Germany; 9 School of Nursing, Midwifery and Health Systems, University College Dublin, Dublin, Ireland; 10 Health Sciences Centre, University College Dublin, Dublin, Ireland; 11 School of Nursing, University of Pennsylvania, Philadelphia

## Abstract

**Importance:**

Physician burnout and retention problems are threatening workforce stability and patient care.

**Objective:**

To compare physician well-being in multiple European countries and the US to inform physician well-being interventions and evaluate whether modifiable aspects of hospital care environments are associated with physician job outcomes.

**Design, Setting, and Participants:**

This cross-sectional study was conducted at 49 hospitals in 6 European countries (Belgium, England, Germany, Ireland, Sweden, and Norway) and 56 US hospitals. European data from physicians and nurses were collected in 2023; US data were collected in 2021. Data were analyzed from February through August 2025.

**Exposures:**

Three explanatory variables were measured at the hospital level: nurse staffing adequacy, clinical care environment, and physician and nurse teamwork.

**Main Outcomes and Measures:**

Primary outcomes include individual-level measures of physician well-being and job outcomes (eg, high burnout, job dissatisfaction, intention to leave employer, and willingness to recommend employer).

**Results:**

Among a total of 21 396 physicians and nurses, 1149 European physicians (mean [SD] age, 41.3 [10.6] years; 536 female [46.5%] and 609 male [52.9%]) and 5334 US physicians (mean [SD] age, 44.5 [11.8] years; 1861 female [34.9%] and 2373 male [44.5%]) reported on their well-being and 3044 European nurses and 11 869 US nurses reported on hospital care environments. Poor well-being was common among hospital physicians in the US and Europe; for example, 324 of 1083 physicians in Europe with responses to the question (29.9%) and 1178 of 4959 physicians with responses to the question (23.8%) intended to leave their job within a year. Improvements in nurse staffing adequacy, clinical care environments, and clinician teamwork were associated with favorable physician job outcomes in the US and Europe. For example, among US hospitals, a 10% increase in favorable care environments was associated with lower odds of physicians intending to leave (odds ratio [OR], 0.78; 95% CI, 0.68-0.90), not recommending their hospital (OR, 0.75; 95% CI, 0.61-0.92), experiencing high burnout (OR, 0.90; 95% CI, 0.83-0.98), and having job dissatisfaction (OR, 0.81; 95% CI, 0.69-0.95). Among European hospitals, a 10% increase in hospital-level reports of nurse staffing adequacy was associated with lower odds of physicians intending to leave (OR, 0.80; 95% CI, 0.71-0.91), not recommending their hospital (OR, 0.73; 95% CI, 0.61-0.88), reporting high burnout (OR, 0.88; 95% CI, 0.78-0.99), and reporting job dissatisfaction (OR, 0.85; 95% CI, 0.73-0.98).

**Conclusions and Relevance:**

In this study, improved hospital nurse work environments were associated with more favorable physician outcomes, and modifiable features of the hospital environment, including having adequate numbers of direct care nurses and strong physician and nurse teamwork, were associated with greater physician well-being. These findings may inform the development and implementation of evidence-based interventions to improve physician well-being and retention.

## Key Points

**Question:**

Does hospital physician well-being vary across European countries and the US, and what modifiable aspects of the hospital care environment are associated with well-being?

**Findings:**

In this cross-sectional study among 21 396 physicians and nurses, indicators of poor well-being were high among physicians in Europe and the US. Physician outcomes were better in hospitals with adequate nurse staffing, supportive care environments, and effective interdisciplinary teamwork.

**Meaning:**

This study’s findings suggest that a promising solution to the concerning levels of unfavorable physician well-being may be implementation of interventions that improve physician hospital work environments, including attention to adequate nurse staffing, organizational structures to promote interdisciplinary teams, and collaboration between physicians and nurses.

## Teaser

This cross-sectional study investigates physician well-being and the association of hospital care environment aspects with physician job outcomes in 6 European countries and the US.

## Introduction

High levels of burnout and turnover among hospital-based physicians are a growing international problem, contributing to physician shortages that are associated with missed care,^1^ eroded patient safety,^2^ and reduced access to hospital and emergency care.^3^ Most research on physician well-being has been conducted in the US,^4,5^ with few studies including cross-country comparisons^6^ that could provide insights into the design of successful interventions. Moreover, most of this research describes the extent of the problem rather than providing the basis for evidence-based interventions.^7,8^

Traditionally, studies on physician well-being have focused on issues extrinsic to the hospital, such as income concerns, rather than intrinsic ones, like perceptions of personal accomplishment, work-life balance, control over work conditions, engagement in institutional decision-making, and respect from management.^9^ Some research from 2024^6^ and 2023^10^ has associated dysfunctions in the hospital care environment, such as excessive workloads and unresponsive administration, with poor job outcomes for physicians and nurses, including burnout, job dissatisfaction, and intentions to leave the employer. However, evidence to guide the design of interventions to improve physician well-being remains scarce.^11^ A meta-analysis of 38 randomized trials of interventions to reduce physician burnout,^12^ including coaching, education, discussion groups, mindfulness practices, and scheduling, identified many methodological limitations and concluded that it was unlikely that these interventions would produce a clinically meaningful impact.

This study had 2 main objectives. The first was to compare descriptive information about physician well-being across Western Europe (Belgium, England, Germany, Ireland, Sweden, and Norway) and the US to determine whether information can be gained to inform physician well-being interventions. The second was to evaluate whether modifiable aspects of hospital care environments were associated with physician job outcomes in the interest of building evidence-based interventions to improve physician well-being and retention. The data provide informative comparisons of multiple national health care systems: European systems in which physician employment is mostly subject to collective agreements with governments, employer associations, or statutory insurers, vs the US system, where physician employment is largely decentralized and privately funded. An additional innovation in this study is an effort to evaluate whether interventions shown previously to be associated with improved well-being and retention of nurses^13,14^ are also associated with more favorable well-being and retention outcomes for physicians. This finding would contribute to our understanding of which interventions are likely to add the most value to health care organizations.

## Methods

### Design and Data

This cross-sectional study leverages 2 unique surveys of hospital-employed physicians and professional nurses in 105 hospitals in 6 European countries (Magnet4Europe Study) and the US (Clinician Well-Being Study). The study followed the Strengthening the Reporting of Observational Studies in Epidemiology (STROBE) reporting guideline.

The Magnet4Europe Study was a quasiexperimental study of European hospitals testing an intervention to improve hospital work environments to improve clinician well-being and retention.^15^ Clinicians in direct care positions (ie, physicians and professional nurses) in participating hospitals were surveyed in December 2023 via online surveys. Surveys were anonymous. No incentives were provided.

The Clinician Well-being Study used a similar approach, surveying physicians and registered nurses in direct care positions in US Magnet hospitals using online surveys in 2021. Clinicians in adult medical and surgical specialties, including general inpatient units, intensive care units, and emergency departments, received surveys. Responses were anonymous. No incentives were provided. Survey items analyzed in this study were asked in both Magnet4Europe and Clinician Well-being Study surveys. Completion of the survey represented informed consent. This study was approved by research ethics committees at KU Leuven, Belgium, and the University of Pennsylvania and in participating countries through a central or decentralized authority.

### Sample of Physicians

Survey data obtained from physicians working in European and US hospitals were used to describe individual physician job outcomes. Data from 1149 European physicians and 5334 US physicians were analyzed.

### Sample of Hospitals

The European sample of hospitals included 49 hospitals across 6 countries (Belgium, England, Germany, Ireland, Norway, and Sweden). Data from nurse survey respondents were analyzed as informants of the hospital clinical care environment. Individual nurse responses to questions about their hospital environment were aggregated to generate hospital-level measures. European study hospitals had a mean of 62 nurse survey respondents reporting on their hospital environment. In total, data from 3044 European nurses were used to generate data about 49 European hospitals. The US sample included 56 hospitals distributed across 22 states, all of which were Magnet designated (a condition of participation for the Clinician Well-Being Study). Magnet designation is a voluntary program providing organizational certification to hospitals that have met criteria signifying engagement of clinicians in practice and management decisions and favorable nurse well-being and patient outcomes.^16^ The US study hospitals had a mean of 211 nurse survey respondents. Data from 11 869 US nurses were used to generate measures of the hospital environment.

### Variables

#### Physician Outcomes

The following primary outcomes were analyzed at the individual physician-level. Burnout was measured using the Emotional Exhaustion subscale of the Maslach Burnout Inventory, which includes 9 items scored on a 7-point Likert scale.^17^ Consistent with prior work, physicians with scores of 27 or higher were dichotomized as having *high burnout*.^10^ Job dissatisfaction was derived from a single-item question using a 4-point Likert scale.^10^ Physicians with responses of *moderately dissatisfied* or *very dissatisfied* were dichotomized as having job dissatisfaction. Physician intent to leave was derived from an affirmative response to the single-item question: “If possible, would you leave your current hospital within the next year as a result of job dissatisfaction?” Physicians were asked whether they would recommend their hospital to colleagues as a good place to work. Response options of *definitely not* or *probably not* on a 4-point Likert scale were dichotomized as the physician not recommending their hospital.

Secondary physician outcomes included measures of anxiety, depression, overall health, control over workload,^18^ and work-life balance.^19^ The Generalized Anxiety Disorder 2-item scale^20^ and Patient Health Questionnaire 2-item scale^21^ were used to measure anxiety and depression, respectively. Overall health was measured using the global health rating item from the Short Form-8 Health Survey.^22^

#### Explanatory Variables

We evaluated 3 explanatory variables at the hospital level. Data obtained from individual nurses were aggregated among nurses within the same hospitals to generate hospital-level measures of the clinical care environment of each hospital. Nurse staffing adequacy was measured at the hospital level and ranged between 0% and 100% based on the percentage of respondents who reported *strongly agree* or *agree* on a 4-point Likert scale in response to the statement “There are enough nurses to get the work done.” Clinical care environment was measured at the hospital level as a continuous variable ranging between 1 and 4, with lower scores indicating the least favorable environments. In this scale derived from the National Quality Forum–endorsed Practice Environment Scale of the Nursing Work Index, respondents were asked to respond to items, for example, related to whether administration is responsive to clinician concerns, whether the supervisor is a good manager, and whether a clear patient care philosophy pervades the clinical environment. This scale has been well-validated and shown to be associated with nurse well-being and patient outcomes.^23,24^ Favorable interdisciplinary clinician teamwork was measured using the Mini-Z 2.0 item “The degree to which my care team works efficiently together is…”. Response options included: optimal, good, satisfactory, marginal, and poor.^18^ Responses of *optimal* and *good* were coded to indicate favorable interdisciplinary clinician teamwork.

#### Covariates

Covariates were used in analytic models. These included a dummy variable for country and physician demographics (age and sex) and unit type (among European physicians) and specialty (among US physicians).

### Statistical Analysis

Research on clinician job outcomes often faces a methodological issue known as common source bias.^25^ This bias arises when the same clinicians provide information on explanatory and outcome variables, potentially leading to spurious correlations. Our study addresses this concern by using nurses as informants about the hospital care environment to estimate job outcomes reported by physicians. This approach is methodologically sound and theoretically compelling given that nurses, who are closely involved in bedside care, provide validated assessments of the care environment that are associated with various patient outcomes.^26^ We hypothesized that organizational attributes of hospitals reported by nurses, including the clinical care environment, nurse staffing adequacy, and clinician teamwork, would be associated with physician well-being.

We first report physician-level outcomes as numbers and percentages within each country. Variations across hospitals within countries in nurse staffing adequacy, clinical care environment scores, and favorable clinician teamwork are illustrated using bar figures with each country’s hospitals in a different color, along with reported means (ranges). To estimate physician-level outcomes with hospital-level nurse reports on hospital factors, we fit logistic regression models with clustered standard errors to account for clustering of physicians within hospitals and a fixed effect for country (ie, the country variable). Models are fully-adjusted for country and physician characteristics. We fit 1 model for each considered hospital factor. Including them all in the same model was not possible owing to moderate to high correlations between variables (Pearson correlation coefficients, 0.57-0.70). The α level for 2-sided tests for statistical significance was set a priori at .05. Data analyses were conducted in Stata statistical software version 17.0 (StataCorp) between February and August 2025.

We estimated expected reductions in the percentage of physicians reporting poor outcomes if nurse staffing adequacy improved to the 90th percentile in Europe or the 90th percentile in US hospitals. Our estimation focuses on changes in physician outcomes based on improvements in nurse staffing adequacy given that patient-to-nurse staffing ratios are amenable to policy intervention.

## Results

Among a total of 21 396 physicians and nurses, 1149 European physicians (mean [SD] age, 41.3 [10.6] years; 536 female [46.5%] and 609 male [52.9%]) and 5334 US physicians (mean [SD] age, 44.5 [11.8] years; 1861 female [34.9%] and 2373 male [44.5%]) reported on their well-being and 3044 European nurses and 11 869 US nurses reported on hospital care environments. Percentages of hospital-based physicians with poor job outcomes are reported in Table 1 (see eTable 1 in Supplement 1 for numbers of physicans with answers to each question), and physicians characteristics (eTable 2 in Supplement 1) and study hospital characteristics (eTable 3 in Supplement 1) can be found in Supplement 1. While differences in physician outcomes across countries were notable, the primary concern was the overall magnitude of unfavorable job outcomes. Overall, 324 of 1083 physicians in Europe with responses to the question (29.9%) and 1178 of 4959 physicians in the US with responses to the question (23.8%) reported intending to leave their job within a year. Reports of intentions to leave the hospital in the next year due to job dissatisfaction ranged from 30 of 147 physicians with responses to the question (20.4%) in Sweden and Norway to 51 of 117 physicians with responses to the question (43.6%) in Ireland. In the US, 655 of 4971 physicians with responses to the question (13.2%) would not recommend their hospital as a good place to work compared with 42 of 117 physicians with responses to the question (35.9%) in Ireland. High burnout ranged from 34 of 198 physicians with responses to the question (17.2%) in Belgium to 53 of 122 physicians with responses to the question (45.3%) in Ireland. Between 13 of 147 physicians with responses to the question (8.8%) in Sweden and Norway and 29 of 122 physicians with responses to the question (23.8%) in Ireland were likely anxious. From 17 of 198 physicians with responses to the question (8.6%) in Belgium to 26 of 122 physicians with responses to the question (21.3%) in Ireland were likely depressed. Between 41 of 198 physicians with responses to the question (20.7%) in Belgium and 69 of 185 physicians with responses to the question (37.3%) in England reported poor overall health. Job dissatisfaction reports ranged from 755 of 4962 physicians with responses to the question (15.6%) in the US to 47 of 117 physicians with responses to the question (40.2%) in Ireland, and most physicians reported having poor control of their workload (ranging from 2885 of 4487 physicians with responses to the question [64.3%] in the US to 98 of 123 physicians with responses to the question [79.7%] in Ireland) and poor work-life balance (ranging from 1788 of 4745 physicians with responses to the question [37.7%] in the US to 313 of 445 physicians with responses to the question [70.3%] in Germany).

**Table 1.** Percentages of Hospital-Based Physicians With Poor Job Outcomes by Country

Job outcomes	Physicians, No. (%)^a^						
	Belgium (n = 205)	England (n = 197)	Germany (n = 459)	Ireland (n = 137)	Sweden and Norway (n = 151)^b^	Europe (n = 1149)^c^	US (n = 5334)^d^
Intent to leave due to job dissatisfaction	41 (20.9)	51 (28.0)	151 (34.2)	51 (43.6)	30 (20.4)	324 (29.9)	1178 (23.8)
Would not recommend hospital as a good place to work	29 (14.8)	45 (24.7)	134 (30.4)	42 (35.9)	27 (18.4)	277 (25.6)	655 (13.2)
High burnout	34 (17.2)	73 (39.9)	142 (32.0)	53 (45.3)	30 (20.4)	332 (30.5)	1626 (34.1)
Anxious	28 (14.1)	41 (22.2)	71 (16.0)	29 (23.8)	13 (8.8)	182 (16.6)	649 (13.9)
Depressed	17 (8.6)	25 (13.5)	70 (15.7)	26 (21.3)	18 (12.2)	156 (14.2)	414 (8.9)
Poor overall health	41 (20.7)	69 (37.3)	115 (25.8)	45 (37.2)	31 (21.1)	301 (27.5)	1391 (30.0)
Job dissatisfaction	32 (16.3)	62 (34.1)	140 (31.7)	47 (40.2)	28 (19.0)	309 (28.5)	775 (15.6)
Poor control over workload	134 (67.3)	141 (75.4)	332 (74.3)	98 (79.7)	108 (72.5)	813 (73.6)	2885 (64.3)
Poor work-life balance	88 (44.4)	106 (57.9)	313 (70.3)	83 (69.7)	76 (51.7)	666 (61.0)	1788 (37.7)

^a^ Physician characteristics can be found in eTable 2 in Supplement 1. Denominators for each percentage are the number of respondents in that country with answers to the question and can be found in eTable 1 in Supplement 1.

^b^ Physician data in Sweden and Norway are combined given the small numbers of participating hospitals and the need to protect the identity of physicians and hospitals.

^c^ The European mean represents the mean of the 6 European country means.

^d^ The US sample of physicians practice in Magnet hospitals.

The Figure shows distributions of explanatory variables among individual European and US hospitals. They all varied widely, with percentages of nurse respondents reporting the outcome in their hospitals ranging from 5.9% to 77.6% for staffing adequacy, 8.3% to 81.8% for favorable clinician teamwork, and 2.4 to 3.5 for clinical care environment scores.

**Figure.**
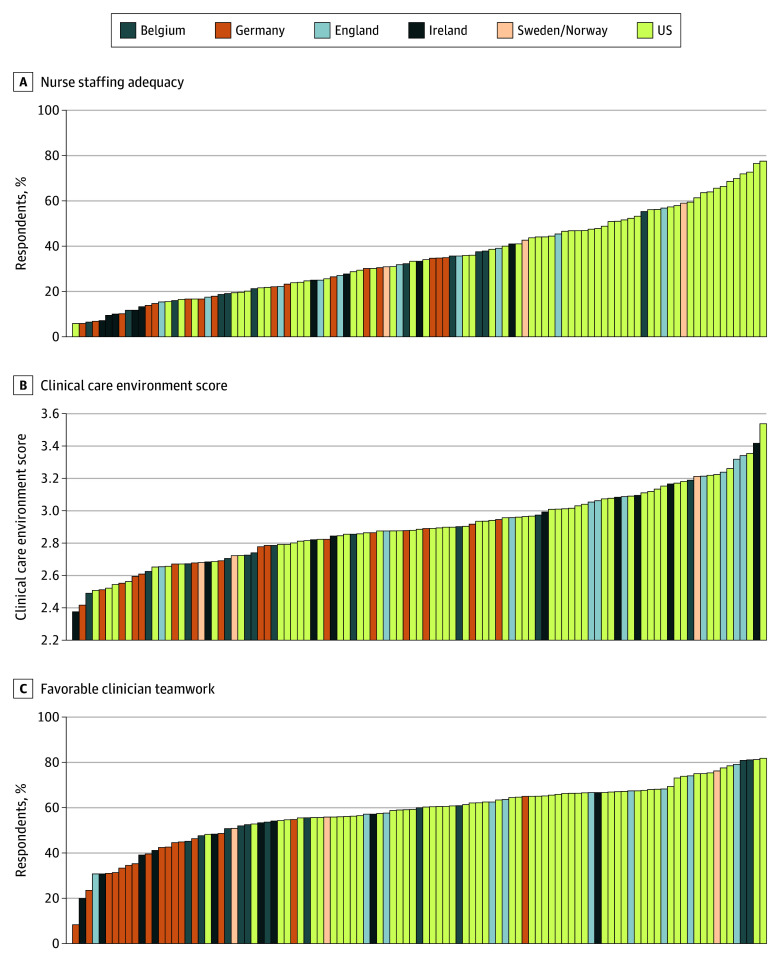
Variation in Modifiable Hospital Factors in 49 European and 56 US Hospitals Variation is shown in nurse staffing adequacy, clinical care environments, and favorable clinician teamwork. Higher values of the clinical care environment score represent more favorable environments.

When aggregated by country (Table 2), less than half of nurses in US hospitals (mean [SD], 43.7% [17.9%]) characterized nurse staffing as adequate, compared with an even lower mean (SD) of 25.7% (13.4%) in Europe. There was considerable variation among European countries; however, nurse staffing adequacy and favorable clinician teamwork were worse overall in Europe compared with the US

**Table 2.** Hospital-Level Variation in Modifiable Hospital Factors

Factor	Mean (SD) [range]						
	Belgium	England	Germany	Ireland	Sweden and Norway	Europe^a^	US
Hospitals, No.	11	10	16	9	3	49	56
Nurses per hospital, mean (SD)	65 (57)	41 (28)	70 (39)	54 (87)	101 (29)	62 (53)	212 (134)
Nurse staffing adequacy, %	26.5 (14.4) [6.5-55.3]	31.6 (13.0) [15.4-56.8]	21.2 (9.9) [5.9-35.0]	19.9 (12.2) [7.1-41.0]	44.2 (14.1) [30.9-59.0]	25.7 (13.4) [5.9-59.0]	43.7 (17.9) [5.9-77.6]
Clinical care environment score^b^	2.8 (0.2) [2.5-3.2]	3.1 (0.2) [2.7-3.3]	2.7 (0.2) [2.4-2.9]	2.9 (0.3) [2.4-3.4]	2.9 (0.3) [2.7-3.2]	2.9 (0.2) [2.4-3.4]	2.9 (0.2) [2.5-3.5]
Favorable clinician teamwork, %	58.2 (12.2) [45.2-81.1]	62.7 (13.1) [30.8-79.2]	39.1 (13.0) [8.3-65.0]	45.6 (14.4) [20.0-66.7]	61.0 (13.4) [50.9-76.2]	50.8 (16.0) [8.3-81.1]	64.1 (7.4) [48.2-81.8]

^a^ The European mean represents the mean of the 6 European country means.

^b^ Clinical care environment score ranges between 1 and 4, with higher numbers being more favorable.

In Europe and the US, we observed reductions in the odds of a physician reporting intentions to leave associated with improvements in nurse staffing adequacy, more favorable clinical care environments, and improvements in clinician teamwork (Table 3). For example, a 10% increase in the hospital percentage of respondents reporting staffing adequacy was associated with a decrease in odds of physician intent to leave that hospital in Europe (odds ratio [OR], 0.80; 95% CI, 0.71-0.91) and the US (OR, 0.91; 95% CI, 0.85-0.97) and a decrease in the odds of a physician being unwilling to recommend their hospital to a colleague (OR, 0.73; 95% CI, 0.61-0.88) in Europe. A 10% improvement to the clinical care environment was also associated with decreases in unwillingness to recommend the hospital in Europe (OR, 0.75; 95% CI, 0.57-0.98) and the US (OR, 0.75; 95% CI, 0.61-0.92); this increase was also associated with lower odds of physicians intending to leave (odds ratio [OR], 0.78; 95% CI, 0.68-0.90), experiencing high burnout (OR, 0.90; 95% CI, 0.83-0.98), and having job dissatisfaction (OR, 0.81; 95% CI, 0.69-0.95) in the US. In Europe, 10% improvements to nurse staffing adequacy (OR, 0.88; 95% CI, 0.78-0.99) and clinician teamwork (OR, 0.89; 95% CI, 0.79-0.99) were associated with lower odds of physician burnout. In the US, improvement in the clinical care environment was associated with reductions in physician burnout. A 10% improvement in nurse staffing adequacy was associated with lower odds of physician job dissatisfaction in Europe (OR, 0.85; 95% CI, 0.73-0.98), and a 1-SD improvement in clinical care environment scores was associated with lower odds of physician job dissatisfaction in the US (OR, 0.81; 95% CI, 0.69-0.95).

**Table 3.** Association of Hospital Factors With Physician Job Outcomes

Factor	Europe^a^		United States^b^	
	OR (95% CI)^c^	*P *value	OR (95% CI)^c^	*P *value
Physicians, No.	1149	NA	5334	NA
Hospitals, No.	49	NA	56	NA
**Intent to leave**				
Nurse staffing adequacy	0.80 (0.71-0.91)	<.001	0.91 (0.85-0.97)	.005
Favorable clinical care environment	0.85 (0.70-1.04)	.11	0.78 (0.68-0.90)	<.001
Favorable clinician teamwork	0.85 (0.76-0.95)	.006	0.78 (0.65-0.94)	.01
**Would not recommend hospital as a good place to work**				
Nurse staffing adequacy	0.73 (0.61-0.88)	<.001	0.90 (0.80-1.00)	.06
Favorable clinical care environment	0.75 (0.57-0.98)	.03	0.75 (0.61-0.92)	.006
Favorable clinician teamwork	0.84 (0.70-1.02)	.07	0.79 (0.61-1.04)	.09
**High burnout**				
Nurse staffing adequacy	0.88 (0.78-0.99)	.03	0.99 (0.95-1.03)	.70
Favorable clinical care environment	0.90 (0.73-1.11)	.34	0.90 (0.83-0.98)	.01
Favorable clinician teamwork	0.89 (0.79-0.99)	.04	0.89 (0.79-1.00)	.06
**Job dissatisfaction**				
Nurse staffing adequacy	0.85 (0.73-0.98)	.03	0.93 (0.86-1.01)	.09
Favorable clinical care environment	0.93 (0.75-1.17)	.54	0.81 (0.69-0.95)	.01
Favorable clinician teamwork	0.89 (0.78-1.03)	.11	0.87 (0.71-1.08)	.209

Abbreviations: NA, not applicable; OR, odds ratio.

^a^ Europe adjusted models include country covariates and physician characteristics (age, sex, and unit type) and clustered standard errors.

^b^ US Clinician Well-Being Study adjusted models include physician characteristics (age, sex, and specialty) and clustered standard errors.

^c^ Models estimate the association of a 10% improvement in nurse staffing adequacy, 1-SD improvement on clinical care environment score, and 10% improvement in clinician teamwork with physician outcomes.

If European and US hospitals improved to the 90th percentile of nurse staffing adequacy (ie, 41.8% of nurses reporting staffing adequacy in Europe and 68.6% of nurses reporting it in the U.S), we estimated associated decreases in physicians reporting intent to leave, with decreases in Europe ranging from 1.4 percentage points in Sweden and Norway to 11.2 percentage points in Ireland and a decrease of 4.3 percentage points in the US (Table 4). In Europe, we estimated decreases ranging from 1.9 percentage points in Sweden and Norway to 13.8 percentage points in Ireland in being unwilling to recommend their hospital, 0.5 percentage points in the US to 6.6 percentage points in Ireland in high burnout, and 1.0 percentage points in Sweden and Norway to 8.1 percentage points in Ireland in job dissatisfaction.

**Table 4.** Estimated Change in Poor Physician Well-Being If Hospitals Improved Nurse Staffing Adequacy to 90th Percentile

Outcome	Physicians^a^					
	Belgium (n = 205)^b^	England (n = 197)^b^	Germany (n = 459)^b^	Ireland (n = 137)^b^	Sweden and Norway (n = 151)^b^	US (n = 5334)^c^
**Intent to leave**						
Observed, No. (%)	41 (20.9)	51 (28.0)	151 (34.2)	51 (43.6)	30 (20.4)	1177 (23.8)
Projected, No. (%)	28.2 (14.4)	44.0 (24.2)	111.9 (25.4)	37.9 (32.4)	28.0 (19.0)	964.3 (19.5)
Change, No. (percentage points)	−12.8 (−6.5)	−7.0 (−3.8)	−39.1 (−8.9)	−13.1 (−11.2)	−2.0 (−1.4)	−212.7 (−4.3)
**Would not recommend**						
Observed, No. (%)	29 (14.8)	45 (24.7)	134 (30.4)	42 (35.9)	27 (18.4)	654 (13.2)
Projected, No. (%)	16.3 (8.3)	36.0 (19.8)	85.1 (19.3)	25.9 (22.1)	24.2 (16.5)	504.4 (10.2)
Change, No. (percentage points)	−12.7 (−6.5)	−9.0 (−5.0)	−48.9 (−11.1)	−16.1 (−13.8)	−2.8 (−1.9)	−149.6 (−3.0)
**High burnout**						
Observed, No. (%)	34 (17.3)	73 (39.9)	142 (32.3)	53 (45.3)	30 (20.5)	1625 (34.1)
Projected, No. (%)	27.7 (14.0)	68.3 (37.3)	120.2 (27.3)	45.2 (38.7)	28.9 (19.8)	1603.5 (33.6)
Change, No. (percentage points)	−6.3 (−3.2)	−4.7 (−2.6)	−21.8 (−5.0)	−7.8 (−6.6)	−1.1 (−0.7)	−21.5 (−0.5)
**Job dissatisfaction**						
Observed, No. (%)	32 (16.3)	62 (34.1)	140 (31.7)	47 (40.2)	28 (19.0)	775 (15.6)
Projected, No. (%)	24.0 (12.2)	56.1 (30.8)	111.4 (25.2)	37.5 (32.1)	26.5 (18.0)	658.9 (13.3)
Change, No. (percentage points)	−8.0 (−4.1)	−5.9 (−3.3)	−28.6 (−6.5)	−9.5 (−8.1)	−1.5 (−1.0)	−116.1 (−2.3)

^a^ Observed values represent actual, non–model-based percentages of physicians experiencing the outcome. Projected values represent model-based estimated percentages of physicians experiencing the outcome and account for physician-level covariates.

^b^ The European analysis is based on the 90th percentile of European hospitals.

^c^ The US analysis is based on the 90th percentile of US hospitals. US estimates for some outcomes (ie, would not recommend, high burnout, and job dissatisfaction) should be interpreted with caution given that there were no associations between nurse staffing adequacy and these physician outcomes in logistic regression models.

## Discussion

In this multicountry cross-sectional study of hospital-based physician job outcomes, we found high levels of physician burnout, job dissatisfaction, and intentions to leave the hospital among 6 European countries and the US. Also common in European countries and the US was significant variation within countries by hospital in poor physician well-being, with some hospitals in each country doing better than others. This outcome suggests that modifiable organizational features of hospitals may be an underexploited and feasible strategy for improving physician well-being. These findings reveal significant threats to attracting and retaining enough physicians to ensure adequate access to hospital services globally. The issues warrant immediate attention and suggest several organizational interventions, feasible in the short-term, that could be associated with improved physician well-being now.

Our study findings highlight modifiable features of hospital care environments as intervention targets for improving physician well-being.^11^ Specifically, we found an association between adequate nurse staffing and supportive clinical care environments that promote interdisciplinary team function with favorable physician outcomes. Physician burnout was more likely in hospitals where respondents reported inadequate nurse staffing. Other organizational features, such as well-functioning interdisciplinary teams and favorable care environments,^27^ were also important to physician well-being. Previous research has documented that greater nurse autonomy, a modifiable feature of hospital work environments, was associated with better functioning interdisciplinary teams.^28^ Most research on clinician well-being focuses on physicians or nurses separately, resulting in competitive solutions in a zero-sum calculation, rather than on how their well-being is intertwined, as shown in our study. This finding suggests that common interventions may benefit physicians and nurses and likely all other health professionals, as well.

Hospital-level measures of nurse staffing adequacy were highly variable in the US, even in Magnet hospitals, which is an important observation worth further consideration among Magnet hospital leaders given our findings of the association between nurse staffing adequacy and the well-being and potential retention of physicians. This new finding about nurse staffing adequacy and physician well-being adds to the substantial literature that nurse staffing adequacy is important to both nurse and patient outcomes.^13^ Many US hospital leaders, including in Magnet hospitals, are instead experimenting with new staffing models that reduce the number of registered nurses despite evidence that such models are potentially dangerous to patients and may contribute to further erosion of clinical well-being and retention.^29^

In the international context, European hospitals were more likely than US hospitals to be considered by their clinicians as understaffed. This may be partially explained by US study hospitals all having Magnet recognition, which is associated with better nurse staffing than non-Magnet designations in the US.^30^ This may explain why nurse staffing adequacy was associated with greater changes in odds of physician outcomes in Europe compared with the US. In contrast, ORs in the association between clinical care environment scores and physician outcomes were larger in the US than in Europe. Thus, while both nurse staffing and the clinical care environment are important, adequate staffing may be a more foundational organizational element associated with clinician outcomes.

The findings are consistent with US research in which 45% of hospital-based physicians indicated that improving nurse staffing levels was a very important organizational intervention likely to reduce their burnout and improve their well-being.^10^ Similarly, 38% of European physicians ranked *improve nurse staffing* as 1 of their top 3 recommended interventions to reduce physician burnout. Having sufficient nurses to get the work done seems to be an essential foundation in the hierarchy of organizational priorities to foster clinician well-being. However, organizational priorities do not generally reflect these empirical results given that organizations are more likely to promote interventions to improve clinician resilience rather than invest in improved working conditions, including nurse staffing adequacy. This disconnect has motivated clinicians in many jurisdictions internationally to seek public policies that require hospital nurse staffing adequacy, as evidenced by policies in Scotland, Wales, Ireland, Australia, Canada, and multiple states in the US.

We estimated that if all European and US study hospitals improved to the 90th percentile of nurse staffing adequacy, there would be an associated decrease in physician burnout ranging from 0.5 percentage points in the US to 6.6 percentage points in Ireland. Other research has shown that even small reductions in physician burnout were associated with meaningful differences in serious medical errors, reductions in work hours, and suicidal ideation.^4,31^ Improvements to the 90th percentile of nurse staffing adequacy in this study is not a particularly high bar for nurse staffing improvement and should be within the capacity of hospitals to achieve given that the 90th percentile in this study was 42% and 69% of nurses reporting staffing adequacy in Europe and the US, respectively.

We observed considerable variation in nurse staffing adequacy, clinical care environments, and clinician teamwork across hospitals within the same European country, suggesting that these differences were not likely associated with policy or resource differences across countries. Even with structural differences, such as public (European) vs private (US) employment models, ensuring sufficient nurse staffing and good work environments are potentially high-leverage strategies for improving physician experiences in both contexts.

This study makes a novel methodological improvement by overcoming common source bias, using nurses as key informants of hospital factors and physicians as informants of their outcomes. Implications of the findings are important given that they suggest that even modest changes in clinical care environments may be associated with improvements in physician and nurse well-being and retention.

### Limitations

This study has several limitations. The study used cross-sectional data, precluding causal inferences about any relationship between hospital environment aspects and physician job outcomes. US study hospitals were Magnet recognized, and European study hospitals had agreed to participate in an intervention to improve their work environments and are not necessarily representative of all hospitals in their countries. Despite this, physician well-being was concerning even in this select group of hospitals. While we documented differences in physician well-being across countries, the most actionable findings concern the significant variation in physician well-being across hospitals within the same countries. This variation suggests that organizational interventions hold promise for associated improvements in physician well-being regardless of country-level differences in health systems. It is possible that the different time frames of data collection (Europe in 2023 and the US in 2021) may have impacted results.

## Conclusions

In this multicountry cross-sectional study of European and US physicians and hospitals, we documented unfavorable physician well-being in every country. These outcomes warrant serious attention, with development and implementation of evidence-based interventions. We identified modifiable features of the hospital clinical care environment, adequate nurse staffing, and supportive interdisciplinary teamwork that were associated with improved physician job outcomes, including lower physician burnout and job dissatisfaction, greater intention to remain with employers, and increased willingness to recruit colleagues. Organizational reforms in hospitals to enhance physician well-being are feasible to implement and have the potential to help retain and sustain a highly qualified international physician workforce.

## Notes

Supplement 1. **eTable 1.** Raw numbers of hospital-based physicians with poor job outcomes by country **eTable 2.** Physician characteristics by country **eTable 3.** Hospital characteristics by country

Supplement 2. **Nonauthor Collaborators.** Magnet4Europe Consortium and US Clinician Well-Being Study Consortium

Supplement 3.
Data Sharing Statement
