# Supplementary material for: Informing Hospital Physician Well-Being Interventions in Europe and the US
Source: JAMA Netw Open. 2025 Nov 17;8(11):e2544067. doi: 10.1001/jamanetworkopen.2025.44067 (PMC12625684; doi:10.1001/jamanetworkopen.2025.44067)
Supplement: Supplement 1. — eTable 1. Raw numbers of hospital-based physicians with poor job outcomes by country eTable 2. Physician characteristics by country eTable 3. Hospital characteristics by country [file jamanetwopen-e2544067-s001.pdf]

Supplemental Online Content

Aiken LH, Sermeus W, McKee M, et al; for the Magnet4Europe Consortium and US Clinician Well-Being Study Consortium. Informing hospital physician well-being interventions in Europe and the US. *JAMA Netw Open*. 2025;8(11):e2544067. doi:10.1001/jamanetworkopen.2025.44067

eTable 1. Raw numbers of hospital-based physicians with poor job outcomes by country

eTable 1. Physician characteristics by country

eTable 2. Hospital characteristics by country

This supplemental material has been provided by the authors to give readers additional information about their work.

© 2025 Aiken LH et al. *JAMA Network Open*.

eTable 1. Raw numbers of hospital-based physicians with poor job outcomes by country

		Physicians, No.						
**Job Outcomes**		***Belgium (n = 205)***	***England (n = 197)***	***Germany (n = 459)***	***Ireland (n = 137)***	***Sweden/ Norway (n = 151)***	**Europe (n = 1,149)**	**U.S. (n = 5,334)**
Intent to leave due to job dissatisfaction	Numerator	41	51	151	51	30	324	1,178
	Denominator	196	182	441	117	147	1,083	4,959
Would not recommend hospital as a good place to work	Numerator	29	45	134	42	27	277	655
	Denominator	196	182	441	117	147	1,083	4,971
High burnout	Numerator	34	73	142	53	30	332	1,626
	Denominator	198	183	444	117	147	1,089	4,774
Anxious	Numerator	28	41	71	29	13	182	649
	Denominator	198	185	445	122	147	1,097	4,657
Depressed	Numerator	17	25	70	26	18	156	414
	Denominator	198	185	445	122	147	1,097	4,669
Poor overall health	Numerator	41	69	115	45	31	301	1,391
	Denominator	198	185	445	121	147	1,096	4,641
Job dissatisfaction	Numerator	32	62	140	47	28	309	775
	Denominator	196	182	441	117	147	1,083	4,962
Poor control over workload	Numerator	134	141	332	98	108	813	2,885
	Denominator	199	187	447	123	149	1,105	4,487
Poor work-life balance	Numerator	88	106	313	83	76	666	1,788
	Denominator	198	183	445	119	147	1,092	4,745

eTable 2. Physician characteristics by country

	Belgium	*England*	*Germany*	*Ireland*	*Sweden/ Norway*	**Europe**	**U.S.**
**No. Physicians**	***205***	***197***	***459***	***137***	***151***	**1,149**	**5,334**
**Age (years)***; mean (SD)*	45.9 (10.1)	38.4 (11.1)	40.6 (10.0)	37.2 (10.1)	44.9 (9.9)		44.5 (11.8)
**Sex;** *No. (%)*							
Male	99 (48.3%)	84 (42.6%)	267 (58.2%)	81 (57.9%)	78 (51.7%)		2373 (44.5%)
Female	105 (51.2%)	112 (56.9%)	188 (41.0%)	59 (42.1%)	72 (47.7%)		1861 (34.9%)
Other	1 (0.5%)	1 (0.5%)	4 (0.9%)	0 (0.0%)	1 (0.7%)		44 (0.08%)
**Unit type**, *No. (%)*							
Surgical unit	52 (25.6%)	39 (20.5%)	131 (28.9%)	20 (15.6%)	46 (30.9%)		--
Internal medicine	57 (28.1%)	62 (32.6%)	98 (21.6%)	62 (48.4%)	48 (32.2%)		--
Intensive care	11 (5.4%)	11 (5.8%)	64 (14.1%)	8 (6.2%)	13 (8.7%)		--
Emergency care	20 (9.9%)	42 (22.1%)	40 (8.8%)	15 (11.7%)	9 (6.0%)		--
Oncology	12 (5.9%)	4 (2.1%)	9 (2.0%)	9 (7.0%)	6 (4.0%)		--
Neurology	20 (9.9%)	2 (1.1%)	30 (6.6%)	2 (1.6%)	3 (2.0%)		--
Geriatrics	12 (5.9%)	11 (5.8%)	6 (1.3%)	3 (2.3%)	2 (1.3%)		--
Other, please specify	19 (9.4%)	19 (10.0%)	75 (16.6%)	9 (7.0%)	22 (14.8%)		--
**Medical specialty,** *No. (%)*							
General Internal Medicine	--	--	--	--	--	--	971 (19.2%)
Internal Medicine, Subspecialty	--	--	--	--	--	--	800 (15.8%)
Oncology	--	--	--	--	--	--	210 (4.2%)
General Surgery	--	--	--	--	--	--	216 (4.3%)
Surgical Subspecialty	--	--	--	--	--	--	556 (11.0%)
Anesthesiology	--	--	--	--	--	--	223 (4.4%)
Critical Care	--	--	--	--	--	--	206 (4.1%)
Emergency Medicine	--	--	--	--	--	--	461 (9.1%)
Rehabilitation Medicine	--	--	--	--	--	--	23 (0.5%)
Psychiatry	--	--	--	--	--	--	50 (1.0%)
Obstetrics / Gynecology	--	--	--	--	--	--	128 (2.5%)

© 2025 Aiken LH et al. *JAMA Network Open*.

Radiology	--	--	--	--	--	--	88 (1.7%)
Pediatrics							98 (1.9%)
Family Practice							188 (3.7%)
Other							839 (16.6%)

Notes. Physician data in Sweden and Norway are combined given the small numbers of participating hospitals and the need to protect the identity of the physicians and the hospitals. European average represents the average of the six European country averages. U.S. sample of physicians practice in Magnet hospitals

© 2025 Aiken LH et al. *JAMA Network Open*.

eTable 3. Hospital characteristics by country

	Belgium	*England*	*Germany*	*Ireland*	*Sweden/ Norway*	**Europe**	**U.S.**
**No. Hospitals**	11	10	16	9	3	**49**	**56**
**Beds,** *median*	*811*	*849*	*809*	*600*	*844*	777	431
(SD, range)	*(624, 267-2202)*	*(265, 397-1389)*	*(582, 196-2042)*	*(218, 340-1010)*	*(556, 288-1400)*	(489, 196-2202)	(328, 72-1452)
**Teaching hospital**, *% (No.)*							
Non-teaching	*81.8% (9)*	*50.0% (5)*	*0% (0)*	*0% (0)*	*33.3% (1)*	30.6% (15)	30.4% (17)
Teaching	*18.2% (2)*	*50.0% (5)*	*100% (16)*	*100% (9)*	*66.7% (2)*	69.4% (34)	69.6% (39)
**Technology capabilities**, *% (No.)*							
Non-high	*72.7% (8)*	*70.0% (7)*	*56.2% (9)*	*22.2% (2)*	*33.3% (1)*	55.1% (27)	25.0% (14)
High	*27.3% (3)*	*30.0% (3)*	*43.8% (7)*	*77.8% (7)*	*66.7% (2)*	44.9% (22)	75.0% (42)

Note. Teaching hospitals were defined by the presence of medical residents and fellows. High technology capabilities was defined as the hospital having the capacity to perform open-heart surgery and/or major organ transplantation.
