# Supplementary material for: Informing Hospital Physician Well-Being Interventions in Europe and the US
Source: JAMA Netw Open. 2025 Nov 17;8(11):e2544067. doi: 10.1001/jamanetworkopen.2025.44067 (PMC12625684; doi:10.1001/jamanetworkopen.2025.44067)
Supplement: Supplement 2. — Nonauthor Collaborators. Magnet4Europe Consortium and US Clinician Well-Being Study Consortium [file jamanetwopen-e2544067-s002.pdf]

| *Group Name(s): Magnet4Europe Consortium, US Clinician Well-Being Study Consortium | | | | | | | |
|---|---|---|---|---|---|---|---|
| ***First Name and Middle Initial(s)** | ***Last Name** | ***Suffix (eg, Jr, III)** | Academic Degrees | Institution | Location (city, state/province, country) | Role or Contribution, eg, chair, principal investigator | Group (if more than 1 Group listed in the byline) and/or Subgroup (eg, Steering Committee) |
| Walter | Sermeus | | | Catholic University Leuven | Belgium | | Magnet4Europe Consortium |
| Hans | De Witte | | | Catholic University Leuven | Belgium | | Magnet4Europe Consortium |
| Wilmar B. | Schaufeli | | | Catholic University Leuven | Belgium | | Magnet4Europe Consortium |
| Simon | Dello | | | Catholic University Leuven | Belgium | | Magnet4Europe Consortium |
| Dorothea | Kohnen | | | Catholic University Leuven | Belgium | | Magnet4Europe Consortium |
| Linda H. | Aiken | | | University of Pennsylvania | USA | | Magnet4Europe Consortium |
| Matthew D. | McHugh | | | University of Pennsylvania | USA | | Magnet4Europe Consortium |
| Herbert | Smith | | | University of Pennsylvania | USA | | Magnet4Europe Consortium |
| Karen B. | Lasater | | | University of Pennsylvania | USA | | Magnet4Europe Consortium |
| Timothy | Cheney | | | University of Pennsylvania | USA | | Magnet4Europe Consortium |
| Douglas | Sloane | | | University of Pennsylvania | USA | | Magnet4Europe Consortium |
| Mary | Del Guidice | | | University of Pennsylvania | USA | | Magnet4Europe Consortium |
| Reinhard | Busse | | | Technical University Berlin | Germany | | Magnet4Europe Consortium |
| Julia | Köppen | | | Technical University Berlin | Germany | | Magnet4Europe Consortium |
| Joan | Kleine | | | Technical University Berlin | Germany | | Magnet4Europe Consortium |

| ***First Name and Middle Initial(s)** | ***Last Name** | ***Suffix (eg, Jr, III)** | Academic Degrees | Institution | Location (city, state/province, country) | Role or Contribution, eg, chair, principal investigator | Group (if more than 1 Group listed in the byline) and/or Subgroup (eg, Steering Committee) |
|---|---|---|---|---|---|---|---|
| Claudia B. | Maier | | | University of Bielefeld | Germany | | Magnet4Europe Consortium |
| Jonathan | Drennan | | | University College Dublin | Ireland | | Magnet4Europe Consortium |
| Vera | McCarthy | | | University College Cork | Ireland | | Magnet4Europe Consortium |
| Elaine | Lehane | | | University College Cork | Ireland | | Magnet4Europe Consortium |
| Noeleen | Brady | | | University College Cork | Ireland | | Magnet4Europe Consortium |
| Ingeborg | Strømseng Sjetne | | | Norwegian Institute of Public Heal | Norway | | Magnet4Europe Consortium |
| Anners | Lerdal | | | Lovisenberg Diaconal Hospital | Norway | | Magnet4Europe Consortium |
| Lars E. | Eriksson | | | Karolinska Institutet | Sweden | | Magnet4Europe Consortium |
| Rikard | Lindqvist | | | Karolinska Institutet | Sweden | | Magnet4Europe Consortium |
| Lisa | Alenius | | | Karolinska Institutet | Sweden | | Magnet4Europe Consortium |
| Ingrid | Svensson | | | Karolinska Institutet | Sweden | | Magnet4Europe Consortium |
| Jane | Ball | | | University of Southampton | England | | Magnet4Europe Consortium |
| Peter | Griffiths | | | University of Southampton | England | | Magnet4Europe Consortium |
| Jackie | Bridges | | | University of Southampton | England | | Magnet4Europe Consortium |
| Jaimie | Ellis | | | University of Southampton | England | | Magnet4Europe Consortium |
| Anne Marie | Rafferty | | | King's College London | England | | Magnet4Europe Consortium |

| *First Name and Middle Initial(s) | *Last Name | *Suffix (eg, Jr, III) | Academic Degrees | Institution | Location (city, state/province, country) | Role or Contribution, eg, chair, principal investigator | Group (if more than 1 Group listed in the byline) and/or Subgroup (eg, Steering Committee) |
|---|---|---|---|---|---|---|---|
| Martin | McKee | | | London School of Hygiene and Tropic | England | | Magnet4Europe Consortium |
| Rachel | Greenley | | | London School of Hygiene and Tropic | England | | Magnet4Europe Consortium |
| Oliver | Sergeant | | | Meplis | Belgium | | Magnet4Europe Consortium |
| Danny | Van Heusden | | | University Hospital Antwerp | Belgium | | Magnet4Europe Consortium |
| Meagan | Cleary | | PhD, BSN, | Advocate Christ Medical Center | Oak Lawn, IL/US | Site PI | U.S. Clinician Well-Being Study Consortium |
| Cathaleen | Ley | | PhD, RN | Anne Arundel Medical Center, Lumin | Annapolis, MD/US | Site PI | U.S. Clinician Well-Being Study Consortium |
| Carla J. | Borchardt | | DNP, RN | Avera McKennan Hospital & Universi | Sioux Falls, SD /US | Site PI | U.S. Clinician Well-Being Study Consortium |
| Jeannine M. | Brant | | PhD, APRN | Billings Clinic | Billings, MT /US | Site PI | U.S. Clinician Well-Being Study Consortium |
| Barbra L | Turner | | DNP, RN | Bon Secours Mercy Health/St. Elizabe | Youngstown, OH /US | Site PI | U.S. Clinician Well-Being Study Consortium |
| Alyssa E. | Leimberger | | MSN, RN | Bon Secours St. Mary's Hospital | Richmond, VA /US | Site PI | U.S. Clinician Well-Being Study Consortium |
| Kristin | Kozlowski | | MSN, RN | Bristol Health | Bristol, CT /US | Site PI | U.S. Clinician Well-Being Study Consortium |
| Bernice L. | Coleman | | PhD, RN | Cedars Sinai Medical Center | Los Angeles, CA /US | Site PI | U.S. Clinician Well-Being Study Consortium |
| Nancy M. | Albert | | PhD, RN | Cleveland Clinic Foundation | Cleveland, Oh /US | Site PI | U.S. Clinician Well-Being Study Consortium |
| Caroline | Stewart | | MSHI, RN | El Camino Health | Mountain View, CA /US | Site PI | U.S. Clinician Well-Being Study Consortium |
| Dinah | Steele | | DNP, RN | Emory Saint Joseph's Hospital | Atlanta, GA/US | Site PI | U.S. Clinician Well-Being Study Consortium |
| Roberta | Kaplow | | PhD, RN | Emory University Hospital | Atlanta, GA /US | Site PI | U.S. Clinician Well-Being Study Consortium |

| *First Name and Middle Initial(s) | *Last Name | *Suffix (eg, Jr, III) | Academic Degrees | Institution | Location (city, state/province, country) | Role or Contribution, eg, chair, principal investigator | Group (if more than 1 Group listed in the byline) and/or Subgroup (eg, Steering Committee) |
|---|---|---|---|---|---|---|---|
| Kathleen | Kaminsky | | MS, RN | Englewood Health | Englewood, NJ /US | Site PI | U.S. Clinician Well-Being Study Consortium |
| Heidi A. | Hinkle | | MSN, RN | Good Samaritan | Vincennes, IN /US | Site PI | U.S. Clinician Well-Being Study Consortium |
| Rocel D. | Besa | | PhD, RN | Hackensack Meridian/Jersey Shore U | Neptune, NJ /US | Site PI | U.S. Clinician Well-Being Study Consortium |
| Kathleen P | Taylor | | DNP, RN | Hackensack Meridian/Raritan Bay Me | Perth Amboy, NJ /US | Site PI | U.S. Clinician Well-Being Study Consortium |
| Kimberly | Dimino | | DNP, RN | Hackensack Meridian/University Mec | Hackensack, NJ /US | Site PI | U.S. Clinician Well-Being Study Consortium |
| Cecelia | Cetnar | | MA, BSN, R | Hackensack Meridian/Riverview Med | Redbank, NJ /US | Site PI | U.S. Clinician Well-Being Study Consortium |
| LS | Leach | | PhD, RN | Huntington Health | Pasadena, CA /US | Site PI | U.S. Clinician Well-Being Study Consortium |
| Sandra L. | Albritton | | MN, BSN, R | Kootenai Hospital | Coeur D Alene, ID /US | Site PI | U.S. Clinician Well-Being Study Consortium |
| Carolyn L. | Davidson | | PhD, RN | Lehigh Valley Hospital | Allentown, PA /US | Site PI | U.S. Clinician Well-Being Study Consortium |
| Timothy | Carrigan | | PhD, RN | Loyola University Medical Center | Maywood, IL /US | Site PI | U.S. Clinician Well-Being Study Consortium |
| Debra A. | Burke | | DNP, MBA | Massachusetts General Hospital | Boston, MA /US | Site PI | U.S. Clinician Well-Being Study Consortium |
| Kristin R. | Anthony | | MSN, RN | Mid Coast Hospital | Brunswick, ME /US | Site PI | U.S. Clinician Well-Being Study Consortium |
| Mildred O. | Kowalski | | PhD, RN | Morristown Medical Center | Morristown, NJ /US | Site PI | U.S. Clinician Well-Being Study Consortium |
| Martha | Rounds | | MS, APRN | Newport Hospital | Newport, RI /US | Site PI | U.S. Clinician Well-Being Study Consortium |
| Jennifer M. | Tudor | | MSN, RN | Northbay Healthcare | Fairfield, CA/ US | Site PI | U.S. Clinician Well-Being Study Consortium |
| Leigh | Griffis | | DNP, RN | Northwell Health/Huntington Hospita | Huntington, NY / US | Site PI | U.S. Clinician Well-Being Study Consortium |

| *First Name and Middle Initial(s) | *Last Name | *Suffix (eg, Jr, III) | Academic Degrees | Institution | Location (city, state/province, country) | Role or Contribution, eg, chair, principal investigator | Group (if more than 1 Group listed in the byline) and/or Subgroup (eg, Steering Committee) |
|---|---|---|---|---|---|---|---|
| Linda M. | Vassallo | | MSN, RN | Northwell Health/Long Island Jewish | New Hyde Park, NY / US | Site PI | U.S. Clinician Well-Being Study Consortium |
| Marie | Mulligan | | PhD, RN | Northwell Health/Mather Hospital | Port Jefferson, NY / US | Site PI | U.S. Clinician Well-Being Study Consortium |
| Irene | Macyk | | PhD, RN | Northwell Health/North Shore Medic | Manhasset, NY / US | Site PI | U.S. Clinician Well-Being Study Consortium |
| Catherine | Manley-Cullen | | MS, RN | Northwell Health/Northern Westche: | Mount Kisco, NY / US | Site PI | U.S. Clinician Well-Being Study Consortium |
| Sandra L. | Hutchinson | | MSN, RN | Northwestern Medicine Delnor Hosp | Geneva, IL/ US | Site PI | U.S. Clinician Well-Being Study Consortium |
| Amanda E. | Haberman | | MSN, RN | Northwestern Medicine/Central DuPa | Winfield, IL / US | Site PI | U.S. Clinician Well-Being Study Consortium |
| Amy L. | Barnard | | MS, APRN | Northwestern Medicine/Lake Forest | Lake Forest, IL / US | Site PI | U.S. Clinician Well-Being Study Consortium |
| Barbara H. | Gobel | | MS, RN | Northwestern Memorial Hospital | Chicago, Il / US | Site PI | U.S. Clinician Well-Being Study Consortium |
| Diana L. | McMahon | | DNP, RN | Ohio State University Comprehensive | Columbus, OH / US | Site PI | U.S. Clinician Well-Being Study Consortium |
| Megan J. | Brown | | MSN, RN | OSF HealthCare Saint Anthony Medic | Rockford, IL / US | Site PI | U.S. Clinician Well-Being Study Consortium |
| Lisa | Strack | | MSN, RN | OSF Healthcare St. Joseph Medical Ce | Bloomington, IL / US | Site PI | U.S. Clinician Well-Being Study Consortium |
| Sheryl A. | Emmerling | | PhD, RN | OSF HealthCare/Saint Francis Medica | Peoria, IL / US | Site PI | U.S. Clinician Well-Being Study Consortium |
| Angela R. | Coladonato | | DNP, RN | Penn Medicine/Chester County Hosp | West Chester, PA / US | Site PI | U.S. Clinician Well-Being Study Consortium |
| Jessie A. | Reich | | PhD, RN | Penn Medicine/Hospital of the Unive | Philadelphia, PA / US | Site PI | U.S. Clinician Well-Being Study Consortium |
| Justin J. | Gavaghan | | BSN, RN | Penn Medicine/Lancaster General Hc | Lancaster, PA / US | Site PI | U.S. Clinician Well-Being Study Consortium |
| James R. | Ballinghoff | | DNP, MBA, | Penn Medicine/Penn Presbyterian M | Philadelphia, PA/ US | Site PI | U.S. Clinician Well-Being Study Consortium |

| ***First Name and Middle Initial(s)** | ***Last Name** | ***Suffix (eg, Jr, III)** | Academic Degrees | Institution | Location (city, state/province, country) | Role or Contribution, eg, chair, principal investigator | Group (if more than 1 Group listed in the byline) and/or Subgroup (eg, Steering Committee) |
|---|---|---|---|---|---|---|---|
| Florence D. | Vanek | | MSN, RN | Penn Medicine/Pennsylvania Hospita | Philadelphia, PA/ US | Site PI | U.S. Clinician Well-Being Study Consortium |
| Karyn A. | Book | | DNP (c), RN | Penn Medicine/Princeton Health | Plainsboro, NJ / US | Site PI | U.S. Clinician Well-Being Study Consortium |
| Kathy | Easter | | MSN, RN | Robert Wood Johnson University Hos | New Brunswick, NJ/ US | Site PI | U.S. Clinician Well-Being Study Consortium |
| Pamela | Duchene | | PhD, APRN | Southwestern Vermont Health Care | Bennington, VT/ US | Site PI | U.S. Clinician Well-Being Study Consortium |
| Mary E. | Lough | | PhD, RN | Stanford Health Care | Stanford, CA / US | Site PI | U.S. Clinician Well-Being Study Consortium |
| Christine L. | Benson | | MSN, RN | Summa Health System Akron Campus | Akron, OH/ US | Site PI | U.S. Clinician Well-Being Study Consortium |
| Maria | Ducharme | | DNP, RN | The Miriam Hospital | Providence, RI / US | Site PI | U.S. Clinician Well-Being Study Consortium |
| Paul | Quinn | | PhD, RN | The Valley Hospital | Ridgewood, NJ/ US | Site PI | U.S. Clinician Well-Being Study Consortium |
| Donna M. | Molyneaux | | PhD, RN | Thomas Jefferson University Hospital | Philadelphia and Gwynedd | Site PI | U.S. Clinician Well-Being Study Consortium |
| Lori | Kennedy | | PhD, RN | UC Davis Health | Sacramento, CA / US | Site PI | U.S. Clinician Well-Being Study Consortium |
| Elizabeth Ellen | Nyheim | | MSN, RN | UC San Diego Health | San Diego, CA / US | Site PI | U.S. Clinician Well-Being Study Consortium |
| Donna M. | Grochow | | MSN, RN | UCI Health | Orange, CA / US | Site PI | U.S. Clinician Well-Being Study Consortium |
| Shannon M. | Purcell | | DNP, RN | University of Alabama at Birmingham | Birmingham, AL/ US | Site PI | U.S. Clinician Well-Being Study Consortium |
| Kirsten | Hanrahan | | DNP | University of Iowa Health Care | Iowa City, IA/ US | Site PI | U.S. Clinician Well-Being Study Consortium |
| Kathy B. | Isaacs | | PhD | UK HealthCare | Lexington, KY/ US | Site PI | U.S. Clinician Well-Being Study Consortium |
| Jill J. | Whade | | MSN, RN | WakeMed Health and Hospitals | Raleigh, NC/US | Site PI | U.S. Clinician Well-Being Study Consortium |
