# Supplementary material for: Informing Hospital Physician Well-Being Interventions in Europe and the US
Source: JAMA Netw Open. 2025 Nov 17;8(11):e2544067. doi: 10.1001/jamanetworkopen.2025.44067 (PMC12625684; doi:10.1001/jamanetworkopen.2025.44067)
Supplement: Supplement 3. — Data Sharing Statement [file jamanetwopen-e2544067-s003.pdf]

Data Sharing Statement

Aiken. Informing Hospital Physician Well-Being Interventions in Europe and the US. *JAMA Netw Open*. Published online November 17, 2025. doi:10.1001/jamanetworkopen.2025.44067

Data
Data available: No
